# Dutch Health Council Advisory Report on Myalgic Encephalomyelitis and Chronic Fatigue Syndrome: Taking the Wrong Turn

**DOI:** 10.3390/diagnostics8020034

**Published:** 2018-05-16

**Authors:** Frank Twisk

**Affiliations:** ME-de-Patiënten Foundation, Zonnedauw 15, 1906 HB Limmen, The Netherlands; frank.twisk@hetnet.nl

**Keywords:** Myalgic Encephalomyelitis, chronic fatigue syndrome, systemic exertion intolerance disease, diagnosis, health policy

## Abstract

Recently, the Dutch Health Council published their advisory report on Myalgic Encephalomyelitis (ME)/Chronic Fatigue Syndrome (CFS) which is meant to determine the medical policy with regard to ME in the Netherlands. The Health Council briefly discusses several diagnostic criteria and proposes to use new diagnostic criteria for “ME/CFS” in research and clinical practice in the future. The advisory report then summarizes organic abnormalities observed in the last decades and concludes that “ME/CFS” is a “serious, chronic, multisystem disease”. According to the Health Council there are no curative treatments for “ME/CFS”, due to lack of knowledge, but specific medication could bring symptomatic relief. The Health Council recommends conducting more research, to (re)educate medical professionals about “ME/CFS”, to appoint three academic expertise centres, which will install a care network for patients, and to fairly judge the limitations (disability) of patients when they apply for a disability income, medical aid and care. The advisory report was welcomed by many patients, because it puts an end to the dominance of the (bio)psychosocial explanatory model and seems to offer a perspective of improving the situation of patients. However, the starting point of the advisory report, a new definition of “ME/CFS”, will have serious (long-lasting) consequences for patients and researchers.

## 1. Terms of Reference for the Dutch Health Council

In the Netherlands, Myalgic Encephalomyelitis (ME) [1,2,3,4] has been considered an alternative label for Chronic Fatigue Syndrome (CFS), as defined by the so-called CFS/Fukuda criteria [5], for decades. As a result of a citizens initiative, which asked to acknowledge that ME and CFS are two distinct diseases, the Dutch Health Council was asked by the Dutch parliament to advise them on the definition of ME and diagnostic criteria; cause(s), course and prevalence of ME; possibilities to prevent and treat the disease; the impact of ME on the patient and on his/her environment and social participation; the organization of treatment of and support for of patients with ME in the Netherlands; and the current scientific developments and perspectives. The advisory report by the Dutch Health Council is meant to determine the medical policy with regard to ME in the Netherlands in the next 5–10 years.

## 2. ME, CFS and Systemic Exertion Intolerance Disease (SEID): Three Distinct Clinical Entities

Although many researchers and clinicians consider ME and CFS to be synonyms, the case criteria for ME and CFS define two distinct clinical entities with partial overlap.

ME [1,2,3,4] is a neuromuscular disease with distinctive muscular symptoms, e.g., prolonged muscle weakness after minimal exertion, paresis and myalgia, symptoms related to neurological disturbances, especially of cognitive, autonomic and sensory functions, variable involvement of cardiovascular and other systems, and a chronic relapsing course. ME was recognized as a clinical entity in 1956 [2] and has been classified as neurological disease by the World Health Organisation since 1969 [6].

Much of the current confusion relating to diagnosis, causes and treatment of ME, originates from the introduction of the name and definition CFS in 1988 [7], as was forecasted by a prominent ME researcher [8]. According to the (re)definition of CFS in 1994 [5] (unexplained) chronic fatigue, must be accompanied by at least four out of a list of eight symptoms, impairment in short-term memory or concentration, a sore throat; tender lymph nodes, muscle pain, multi-joint pain, headaches (new type, pattern, or severity), unrefreshing sleep, and post-exertional “malaise”. As a consequence of the polythetic nature of its definition, the case criteria [5] define a heterogeneous population of patients with chronic fatigue as principle complaint.

**Figure 1:**
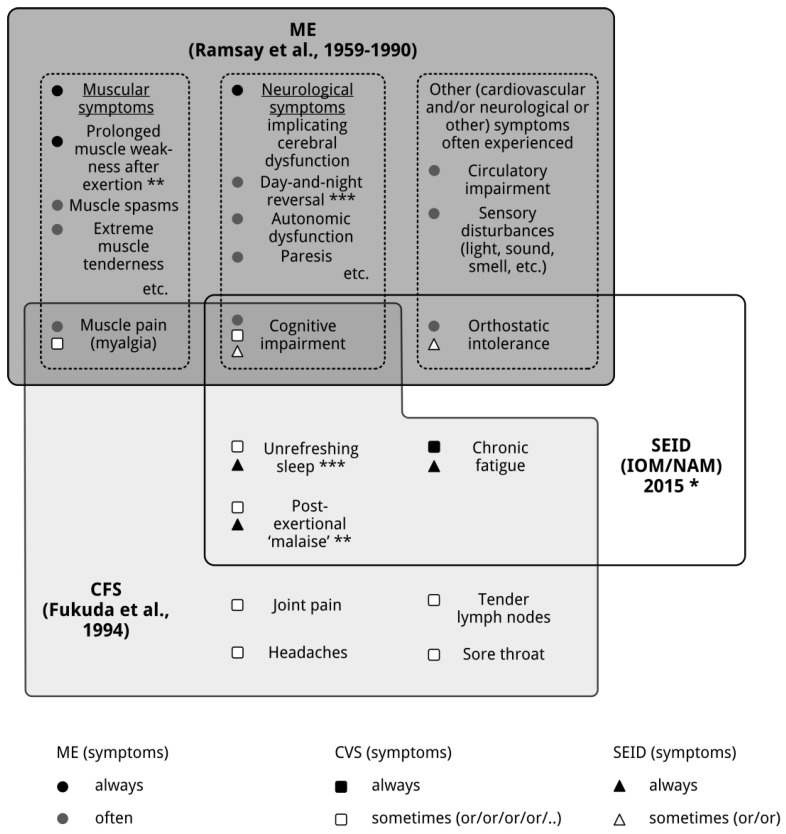
ME, CFS, and SEID: distinct clinical entities. Note: The sizes of the figures do not reflect absolute numbers, but the prevalence of SEID and CFS are considerably higher than that of ME. * Psychological and (other) medical disorders are not excluded; ** Prolonged muscle weakness after exertion is very specific (can be assessed objectively), post-exertional “malaise” is undefined (unspecific); *** Day-and-night-reversal is much more specific than “unrefreshing sleep”. IOM: Institute of Medicine, NAM: National Academy of Medicine.

As a consequence of its case criteria, ME [2,3,4] and CFS [5] are two distinct, partially overlapping, clinical entities. A part of the CFS [5] patient group qualifies as ME [2,3,4] patients, and a ME [2,3,4] patient subgroup meets the diagnostic criteria of CFS [5]. As can be seen in Figure 1, the only common symptoms of ME and CFS are cognitive impairment and myalgia, which are very often experienced by ME [2,3,4] patients, and optional for the diagnosis of CFS [5]. Post-exertional “malaise” (a long-lasting increase of symptoms after physical or mental exertion), an ill-defined, optional symptom for the diagnosis CFS [5], is not equivalent to post-exertional muscle weakness, a very specific symptom mandatory for the diagnosis ME [2,3,4].

In 2015, the US Institute of Medicine (IOM), now called the National Academy of Medicine (NAM), proposed new case criteria and a new name, Systemic Exertion Intolerance Disease (SEID) [9], to replace the diagnoses ME [2,3,4] and CFS [5] and the labels ME and CFS. SEID is defined by chronic fatigue, unrefreshing sleep, and post-exertional “malaise” and orthostatic intolerance and/or cognitive deficits [9]. However, since the case criteria of ME [2,3,4] and CFS [5] define two distinct entities, this is impossible. That’s not a matter of opinion, but a matter of definition [10]. Moreover, introducing a new set of new diagnostic criteria will create even more confusion. Especially since the overlap between, ME [2,3,4], CFS [5] and SEID [9] is relatively small [11]. As can be seen in Figure 1, the only common symptoms of ME [2,3,4] and SEID [9] are cognitive impairment and orthostatic intolerance, often present in ME [2,3,4] and facultative for the diagnosis SEID [9]. The only common symptom of CFS [5] and SEID [9] is chronic fatigue, mandatory for both CFS [5] and SEID [9]. Post-exertional “malaise” and unrefreshing sleep are mandatory for the diagnosis SEID [9] and optional for the diagnosis CFS [5], while cognitive deficits are optional for both CFS [5] and SEID [9]. For that reason, it is not surprising that a recent study [12] found that 25% of the CFS [5] patients in a community epidemiology database did not meet the diagnostic criteria of SEID [9].

## 3. How the Dutch Health Council’s Advisory Report Came into Being

### 3.1. Assignment

The Dutch Council was specifically asked to investigate ME, not CFS [5]: the definition of ME (case criteria), the cause, natural course and prevalence of ME, opportunities to prevent the illness, the impact of ME on the life of patients, his/her relatives and societal participation of patients, the organisation of treatment and support for patients, and the current scientific developments and perspectives [13].

### 3.2. Method

Contrary to the assignment, the Health Council broadened their scope to “ME/CFS”. The Dutch Health Council did not conduct a review of the research into ME (and CFS), but based their findings and definition of “ME/CFS” on four reviews by others: (1) the IOM report [9], which reviewed CFS [5] studies only and proposed new criteria; (2) a systemic review of diagnostic criteria commissioned by the US National Institutes of Health (NIH) [14]; (3) a systemic review of diagnostic criteria and treatments conducted by the Agency for Healthcare Research and Quality (AHRQ) [15]; and (4) a systematic review of treatments for “ME/CFS” conducted by order of the NIH [16]. Importantly these reviews of diagnostic criteria all lacked the original criteria for ME [2,3,4]. Based on these reviews, the Dutch Health Council recommends adopting the SEID criteria to define “ME/CFS” [17]. So, in essence, a name expressing two different clinical entities, ME [2,3,4]/CFS [5], is defined by a third set of case criteria (SEID) [9].

### 3.3. Outcome

Considering the next quote, the Health Council does not acknowledge the existence of the neuromuscular disease ME [2,3,4] as a “real” clinical entity: “The name ‘Myalgic Encephalomyelitis’ has its own disadvantages. ‘Myalgia’, muscle pain, is not a typical symptom, ‘encephalomyelitis’ suggest inflammation of the nervous system, while it is unclear if, and if so, how inflammation of the brain plays a role” [17]. This quote affirms that the Health Council not only rejects the label ME, but also its definition. Instead the Health Council proposes to use a new, non-validated definition of “ME/CFS” as described by the IOM (SEID) [9] in research and in clinical practice. As explained, this definition of “ME/CFS” does not fully describe ME.

The report briefly reviews various organic abnormalities. Based on this review, the Heath Council concludes that “ME/CFS” is “a serious, chronic multi-systemic disease” [17]. The Health Council concludes that, due to the fact that the cause(s) of “ME/CFS” is (are) unknown, there are no curative therapies available, and suggest some potential therapies for symptomatic relief, including sleep and pain medicine.

The Health Council also made four recommendations: (1) to conduct more research into organic abnormalities, (2) to install academic expertise centres for “ME/CFS”, which implement and coordinate a care network for patients, (3) to (re)educate medical professionals and other care takers about “ME/CFS” and (4) medical disability assessors to recognize the serious functional limitations of ‘ME/CFS’ patients [17].

## 4. Due to the New Definition of “ME/CFS” the Four Recommendations Will Have Negative Consequences for Patients with ME and Patients with CFS

ME and CFS are two different notions, which cannot be replaced by a third notion [10]. The Health Council should have made it clear that the case criteria for ME [2,3,4] and CFS [5] define two distinct clinical entities [11]. Instead the Health Council proposes a third definition: “ME/CFS”. Using a new definition of “ME/CFS” [9] in research and clinical practice has profound negative consequences on the outcome of the recommendations, which will be discussed in the next paragraphs.

“ME/CFS” as proposed by the Health Council (SEID) [17] is an ill-defined concept based on five abstract notions (symptoms): chronic fatigue, unrefreshing sleep, post-exertional “malaise”, orthostatic intolerance, and cognitive impairment. Psychological disorders which could account for the symptoms are not excluded.

Due to the definition, substantial subgroups of patients with well-known medical diseases and psychological disorders also qualify as “ME/CFS” patient in the future. A study [12] found that 33% of the patients with MS, 47% of patients with Lupus, and 27% of patients with major depressive disorder met the case criteria of “ME/CFS” [9]. Not only patients with other medical diseases and psychological disorders, but a substantial number of patients with ME [2,3,4] and CFS [5] will be misdiagnosed also using the new definition of “ME/CFS”. This is illustrated by the finding in the same study [12] that 25% of the CFS [5] patients did not qualify as a “ME/CFS” patient [9].

### 4.1. The Consequences of the Wrong Definition for Research into ME

The first recommendation of the Health Council [17] is to conduct more research into organic abnormalities. Although this recommendation is commendable, taking the case criteria of “ME/CFS” [9] as a starting point for research is not the right choice.

**Figure 2:**
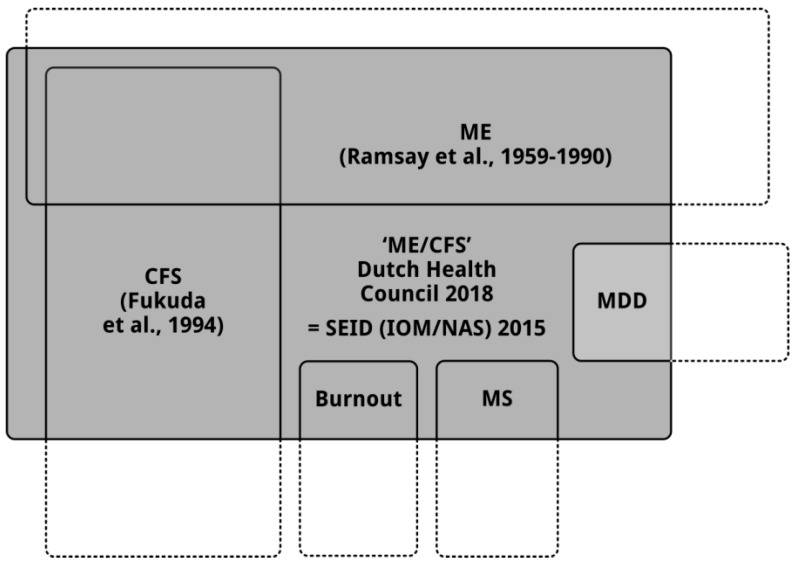
“ME/CFS” (SEID) excludes ME and CFS patient subgroups and includes patient subgroups with other medical diseases and psychological conditions. MDD: Major Depressive Disorder, MS: Multiple Sclerosis.

As can be seen in Figure 2, the diagnostic criteria for “ME/CFS” [9] exclude ME [2,3,4] and CFS [5] patient subgroups and include patients with other diseases, e.g., MS, mitochondrial disease, and burnout, and psychological disorders, e.g., depression. When you investigate an ill-defined, heterogeneous patient group [10,12], the risk of finding no significant differences, e.g., due to data smoothing, increases drastically. Even more, if certain other patient groups meeting the case criteria for “ME/CFS” [9], e.g., people with burnout [18] or patients with major depressive disorder (MDD), are over-represented in a study, a study can draw the wrong conclusions. For example, when looking at to the hypothalamic-pituitary-adrenal (HPA) axis, hypercortisolism was found in 40–60% of drug-free MDD patients [19], while hypocortisolism [20] and blunted cortisol responses [21] have been observed in ME/CFS subgroups, and HPA-axis functioning in clinically diagnosed burnout patients seems to be normal [22].

The heterogeneity of the CFS patient group [23] due to its vague definition, is one of the important reasons why research into CFS [5] has not been very effective, yielding positive findings, no significant differences and contradictive results in CFS [9,24]. So, research into patients with “ME/CFS” [9], an even more heterogonous patient population than CFS [5], most likely will not yield any significant findings and discriminative abnormalities, which will “confirm” the incorrect perception [9] that ME [2,3,4], CFS [5], “ME/CFS” (SEID) [9] are “functional somatic syndromes” [25]. Since patients with other diseases, like burnout, and psychic disorders, e.g., major depression, could respond to cognitive behavioural therapy (CBT) and graded exercise therapy (GET), the controversy [26,27,28] with regard to the effect and safety of CBT and/or GET for ME [2,3,4] and CFS [5] will likely perpetuate.

Even when results of studies are stratified by case criteria for ME [2,3,4] and CFS [5], as proposed, findings and conclusions cannot be generalized to ME or CFS, since a group of patients with ME and CFS does not qualify as being a “ME/CFS” patient [12].

In conclusion, due to the patients studied, research into the organic abnormalities in “ME/CFS” [9] most likely will yield no significant results or contradictory results. Moreover, introducing a new definition of “the” disease implicates that the outcomes of all existing research studies into ME [2,3,4] (1936–1990) and/or CFS [5] (1988–2018), more than 7700 studies in the PubMed data base, will lose their scientific significance.

### 4.2. The Consequences of the Wrong Definition for the Scope of Academic Expertise Centers and Instalment of a Care Network for Patients with ME and Patients with CFS

The consequences of using a wrong definition of ME [2,3,4] for research conducted by the future academic expertise centres have been addressed to in the previous paragraph. This paragraph focuses on access to care, diagnosis and treatment.

Patients report difficulties in gaining access to medical care [29] and the vast majority of patients are dissatisfied with the quality of care received [30]. A study concluded: “Dissatisfied patients were significantly more likely to describe delay, dispute or confusion over diagnosis; to have received and rejected a psychiatric diagnosis; to perceive doctors as dismissive, sceptical or not knowledgeable about CFS [..]” [31]. Note that the label CFS in this quote refers to ME and/or CFS.

Instalment of a care network and outpatient clinics at academic hospitals based for “ME/CFS” [9] has several implications. As explained, a part of the ME [2,3,4] and CFS [5] patient group will not be diagnosed as “ME/CFS” [9] patient, and therefore will have difficulties to have access to the medical care supplied by the care network. People with other medical diseases, e.g., MS, and psychological condition are at risk of being misdiagnosed as “ME/CFS” [9] patient. This risk of misdiagnosis is illustrated by the observation [32] that 8.8% of patients with mitochondrial disease were first diagnosed as being CFS [5] patients. Due to its heterogeneity, the group of patients with ME [2,3,4], CFS [5] and various other medical and psychological diseases meeting the diagnosis “ME/CFS” are at risk of not receiving appropriate care. Most importantly patients with severe ME [2,3,4], which are bedbound or housebound, will likely experience large difficulties to get access to proper medical care.

All in all, using new case criteria [9] to define the “ME/CFS” patient group increases the risk of excluding patients with ME [2,3,4] and/or CFS [5] and misdiagnosis and inappropriate treatment of people with medical diseases and psychiatric disorders. Access to and satisfaction with medical care likely will not improve substantially, which, looking at the current dissatisfaction of patients, is a missed opportunity.

### 4.3. The Consequences of a Wrong Definition of ME for the (Re)education of Medical Professionals and Care Takers

Another recommendation of the Dutch Health Council [17] is to (re)educate medical professionals and other care takers about the “serious chronic disease” “ME/CFS”. While the intention of the advice is commendable, if medical professionals and care takers are not (re)educated properly, it will have no effect or even a negative effect.

“ME/CFS” [9] does not do justice to the nature of ME [2,3,4], which is a neuromuscular disease, not a “fatigue syndrome”. In the case of ME [2,3,4], history seems to repeat itself. The ill-defined notion “ME/CFS” [9] will create a wrong perception of ME [2,3,4], just like CFS [5] did in the last decades. This is illustrated by a survey from 2011 [33] which found that 84% of the responding members of Association of British Neurologists did not consider CFS [5] to be a neurological condition, despite the fact that the World Health Organisation (WHO) has acknowledged ME as a neurological disease since 1969 [5] and considers the labels CFS and ME to be exchangeable since 1992 [34].

ME [2,3,4] and CFS [5] are two different diagnoses by definition. When (re)educating medical professionals and care takers, it should be made clear that ME [2,3,4] is not a “fatigue syndrome”/CFS [5], but a distinct a neuromuscular disease. Replacing ME [2,3,4] and CFS [5] by “ME/CFS” [9], with a new definition, does not solve problems, but rather adds to the current confusion with regard to diagnosis and treatment [35].

Due to its chronic fatigue-cantered definition [36] and its name [37], patients with CFS [5], part of which qualifies as ME [2,3,4] patient, experience significant stigma [38]. The label “ME/CFS” is inappropriate and its fatigue-cantered definition will not reduce the stigma attached to ME [2,3,4] nor will it change the wrong perception. This is illustrated by media coverage of the release of the report of the Health Council [17], reflected in headings like “Chronic fatigue remains enigmatic” [39] and “Acknowledgement for ME patients: Chronic fatigue classified as serious illness” [40].

### 4.4. The Consequences of a Wrong Definition of ME (and CFS) and Not Using Objective Tests for the Rights to~Receive a Disability Income, Care and Medical Aid

The existence and magnitude of various characteristic symptoms of ME [2,3,4] and CFS [5] can be assessed objectively [41,42]. For example, loss of muscle power and prolonged muscle weakness after physical exertion [43] can be established by measuring muscle power during repeated contractions using dynamometers with 24 h rest in-between. Cognitive deficits, e.g., impairments in information processing speed, (working) memory, reaction time, and sustained attention, “can be identified if appropriate measures are used” [44]. Post-exertional “malaise” can be assessed objectively by comparing performance indicators (maximum workload, maximum oxygen uptake, anaerobic threshold and corresponding oxygen uptake) of two cardiopulmonary exercise tests with 24 h in-between [45] and comparing cognitive performance before and after a cardiopulmonary exercise test [46]. Orthostatic intolerance can be reflected by postural orthostatic tachycardia and instantaneous/delayed orthostatic hypotension during tilt table or active standing tests [47,48], and/or disequilibrium established by neurological tests, e.g., tandem gait test, standing on one leg test, and the Romberg test [49]. Sitting intolerance can be reflected by tachycardia and/or hypotension during an active sitting test [49].

Two of the three core symptoms of “ME/CFS” [9], fatigue and unrefreshing sleep, are ill-defined symptoms, which cannot be assessed objectively. Various aspects of post-exertional “malaise”, the third mandatory symptom, can be “quantified” [42]. This implies that the diagnosis “ME/CFS” [9] will largely be dependent on self-report.

Mainly as a consequence of “the misconception that it is a psychogenic illness or even a figment of the patient’s imagination” [9] patients “continue to struggle to have their condition recognised as disabling in the face of public and professional prejudice and discrimination” [50], despite often being severely disabled [50,51], as acknowledged by the Dutch Health Council [17]. Due to their disability patients work fewer hours and have lower incomes, e.g., when compared to MS patients [50], and have substantially higher direct medical costs [52]. Looking at the scepticism among medical professionals, insurance physicians and occupational health experts [9], it is crucial that relevant symptoms can be assessed objectively. Introducing a hybrid diagnosis, “ME/CFS” [9], which does not do justice to both ME [2,3,4] and CFS [5], and is defined by three abstract symptoms, based on self-report, does not help patients. Looking at the negative response of Dutch Association of medical insurance professionals to the advisory report of the Health Council [53], patients will be stuck in the middle.

## 5. Discussion

The purpose of the Health Council advisory report is to effect a change of course, which is commendable. While the Health Council acknowledges that “ME/CFS” is “a serious chronic multisystemic disease” and makes important recommendations to improve the situation for patients in The Netherlands, these recommendations likely turn out to be counterproductive when using the wrong definition of ‘ME/CFS’ [17].

This definition does not accurately reflect the original definition of ME [2,3,4]. One might argue that the Dutch Health Council, instead of introducing a new definition of “ME/CFS” [9] or embracing the original criteria for ME [2,3,4], should have adopted the International Consensus Criteria for ME [54], which are meant to replace the Canadian Consensus Criteria for ME/CFS [55]. However, as long as the International Consensus Criteria for ME (ME-ICC) [54] are not validated and the discrepancies between ME [2,3,4] and ME-ICC [55] are not resolved, replacing ME [2,3,4] by ME-ICC [55] is not a good alternative (yet).

One might also argue that the positive elements of the advisory report [17] (recognition of the suffering of patients and a change of course) outweigh the use of the new definition of “ME/CFS”, but the problem is that the definition of “ME/CFS” largely determines the outcome of the recommendations. Good intentions can turn out negatively just due to a wrong definition.

## 6. Conclusions

As argued before, replacing ME [2,3,4] and CFS [5], two distinct clinical entities, by a new clinical entity with ill-defined symptoms, “ME/CFS” (SEID) [9], is not a step forward [10]. Taking a wrong definition of “the” disease as a starting point [17], the four recommendations will have serious negative consequences for patients with ME [2,3,4], and patient with CFS [5], despite all good intentions of the Health Council.

## References

[1] Acheson E.D. (1959). The clinical syndrome variously called benign myalgic encephalomyelitis, Iceland disease and epidemic neuromyasthenia. Am. J. Med..

[2] Acheson D.E. (1956). A new clinical entity?. Lancet.

[3] Ramsay A.M., Dowsett E.G., Hyde B.M., Goldstein J., Levine P. (1992). Myalgic Encephalomyelitis: Then and now. The Clinical and Scientific Basis of Myalgic Encephalomyelitis/Chronic Fatigue Syndrome.

[4] Dowsett E.G., Ramsay A.M., McCartney R.A., Bell E.J. (1990). Myalgic Encephalomyelitis—A persistent enteroviral infection?. Postgrad. Med. J..

[5] Fukuda K., Straus S.E., Hickie I., Sharpe M., Dobbins J.G., Komaroff A.L. (1994). The chronic fatigue syndrome: A comprehensive approach to its definition and study. Ann. Intern. Med..

[6] World Health Organization (1967). International Classification of Diseases, Eighth Revision (ICD-8).

[7] Holmes G.P., Kaplan J.E., Gantz N.M., Komaroff A.L., Schonberger L.B., Straus S.E., Jones J.F., Dubois R.E., Cunningham-Rundles C., Pahwa S. (1988). Chronic fatigue syndrome: A working case definition. Ann. Intern. Med..

[8] Dowsett E.G. (1988). Myalgic encephalomyelitis, or what?. Lancet.

[9] Institute of Medicine (2015). Beyond Myalgic Encephalomyelitis/Chronic Fatigue Syndrome: Redefining an Illness.

[10] Twisk F.N.M. (2016). Replacing Myalgic Encephalomyelitis and chronic fatigue syndrome with systemic exercise intolerance disease is not the way forward. Diagnostics.

[11] Twisk F.N.M. (2018). Myalgic Encephalomyelitis, Chronic Fatigue Syndrome, and Systemic Exertion Intolerance Disease: Three distinct clinical entities. Challenges.

[12] Jason L.A., Sunnquist M., Kot B., Brown A. (2015). Unintended consequences of not specifying exclusionary illnesses for systemic exertion intolerance disease. Diagnostics.

[13] Tweede Kamer der Staten-Generaal (Dutch Parliament) (2015). Adviesaanvraag van de Tweede Kamer aan de Gezondheidsraad (Request of Advice by the Dutch Health Council). https://www.gezondheidsraad.nl/sites/default/files/grpublication/adviesaanvraag_myalgische_encefalomyelitis.pdf.

[14] Haney E., Smith M.E., McDonagh M., Pappas M., Daeges M., Wasson N., Nelson H.D. (2015). Diagnostic methods for Myalgic Encephalomyelitis/chronic fatigue syndrome: A systematic review for a National Institutes of Health Pathways to Prevention workshop. Ann. Intern. Med..

[15] Smith M.E.B., Nelson H.D., Haney E., Pappas M., Daeges M., Wasson N., McDonagh M. (2014). Diagnosis and Treatment of Myalgic Encephalomyelitis/Chronic Fatigue Syndrome.

[16] Smith M.E., Haney E., McDonagh M., Pappas M., Daeges M., Wasson N., Fu R., Nelson H.D. (2015). Treatment of Myalgic Encephalomyelitis/chronic fatigue syndrome: A systematic review for a National Institutes of Health Pathways to Prevention Workshop. Ann. Intern. Med..

[17] Gezondheidsraad (2018). ME/CVS. https://www.gezondheidsraad.nl/sites/default/files/grpublication/kernadvies_me_cvs_1.pdf.

[18] Verschuren C.M., Nauta A.P., Bastiaanssen M.H.H., Terluin B., Vendrig A.A., Verbraak M.J.P.M., Flikweert S., Vriezen J.A., van Zanten-Przybysz I., Loo M.A.J.M. (2011). Richtlijn één lijn in de Eerste lijn bij Overspanning en Burnout. Multidisciplinaire Richtlijn Overspanning en Burnout voor Eerstelijns Professionals (Multidisciplinary Guideline for Being Overstrained and Burnout for Front-line Professionals). https://www.nvab-online.nl/sites/default/files/bestanden-webpaginas/MDRL_Overspanning-Burnout.pdf.

[19] Parker K.J., Schatzberg A.F., Lyons D.M. (2003). Neuroendocrine aspects of hypercortisolism in major depression. Horm. Behav..

[20] Tomas C., Newton J., Watson S. (2013). A review of hypothalamic-pituitary-adrenal axis function in chronic fatigue syndrome. ISRN Neurosci..

[21] Van Den Eede F., Moorkens G., Hulstijn W., Van Houdenhove B., Cosyns P., Sabbe B.G., Claes S.J. (2008). Combined dexamethasone/corticotropin-releasing factor test in chronic fatigue syndrome. Psychol. Med..

[22] Mommersteeg P.M., Heijnen C.J., Verbraak M.J., van Doornen L.J. (2006). Clinical burnout is not reflected in the cortisol awakening response, the day-curve or the response to a low-dose dexamethasone suppression test. Psychoneuroendocrinology.

[23] Wilson A., Hickie I., Hadzi-Pavlovic D., Wakefield D., Parker G., Straus S.E., Dale J., McCluskey D., Hinds G., Brickman A. (2001). What is chronic fatigue syndrome? Heterogeneity within an international multicentre study. Aust. N. Z. J. Psychiatry.

[24] Twisk F.N.M. (2014). The status of and future research into Myalgic Encephalomyelitis and chronic fatigue syndrome: The need of accurate diagnosis, objective assessment, and acknowledging biological and clinical subgroups. Front. Physiol..

[25] Wessely S., Nimnuan C., Sharpe M. (1999). Functional somatic syndromes: One or many?. Lancet.

[26] White P.D., Goldsmith K.A., Johnson A.L., Potts L., Walwyn R., DeCesare J.C., Baber H.L., Burgess M., Clark L.V., Cox D.L. (2011). Comparison of adaptive pacing therapy, cognitive behaviour therapy, graded exercise therapy, and specialist medical care for chronic fatigue syndrome (PACE): A randomised trial. Lancet.

[27] Wilshire C., Kindlon T., Matthees A., McGrath S. (2017). Can patients with chronic fatigue syndrome really recover after graded exercise or cognitive behavioural therapy? A critical commentary and preliminary re-analysis of the PACE trial. Fatigue.

[28] Twisk F.N.M. (2018). Graded exercise therapy for chronic fatigue syndrome in GETSET. Lancet.

[29] Sunnquist M., Nicholson L., Jason L.A., Friedman K.J. (2017). Access to medical care for individuals with Myalgic Encephalomyelitis and chronic fatigue syndrome: A call for centers of excellence. Mod. Clin. Med. Res..

[30] De Kimpe A., Crijnen B., Kuijper J., Verhulst I., van der Ploeg Y. (2016). Zorg voor ME—Enquête onder ME-Patiënten naar Hun Ervaringen met de Zorg in Nederland 2016 (Care for ME—Survey of ME Patients about Their Experiences with Health Care in The Netherlands, 2016).

[31] Deale A., Wessely S. (2001). Patient’s perceptions of medical care in chronic fatigue syndrome. Soc. Sci. Med..

[32] Grier J., Hirano M., Karaa A., Shepard E., Thompson J.L.P. (2018). Diagnostic odyssey of patients with mitochondrial disease: Results of a survey. Neurol. Genet..

[33] Wojcik W., Armstrong D., Kanaan R. (2011). Chronic fatigue syndrome: Labels, meanings and consequences. J. Psychosom. Res..

[34] World Health Organization (1992). International Classification of Diseases, Tenth Revision (ICD-10).

[35] Holgate S.T., Komaroff A.L., Mangan D., Wessely S. (2011). Chronic fatigue syndrome: Understanding a complex illness. Nat. Rev. Neurosci..

[36] Shlaes J.L., Jason L.A., Ferrari J.R. (1999). The development of the Chronic Fatigue Syndrome Attitudes Test. A psychometric analysis. Eval. Health Prof..

[37] Jason L.A., Taylor R.R., Plioplys S., Stepanek Z., Shlaes J. (2002). Evaluating attributions for an illness based upon the name: Chronic fatigue syndrome, myalgic encephalopathy and Florence Nightingale disease. Am. J. Community Psychol..

[38] Looper K.J., Kirmayer L.J. (2004). Perceived stigma in functional somatic syndromes and comparable medical conditions. J. Psychosom. Res..

[39] Vollebregt B. (2018). Chronische Vermoeidheid Blijft Raadselachtig. https://www.trouw.nl/samenleving/chronische-vermoeidheid-blijft-raadselachtig~a516cbdc.

[40] RTL Nieuws (2018). Erkenning voor ME-patiënt: Chronische Vermoeidheid Aangemerkt als Ernstige Ziekte. https://www.rtlnieuws.nl/nederland/erkenning-voor-me-patient-chronische-vermoeidheid-aangemerkt-als-ernstige-ziekte.

[41] Twisk F.N.M. (2015). Accurate diagnosis of myalgic encephalomyelitis and chronic fatigue syndrome based upon objective test methods for characteristic symptoms. World J. Methodol..

[42] Twisk F.N.M. (2015). Prolonged abnormal effects of exercise in Myalgic Encephalomyelitis and chronic fatigue syndrome. Jacobs J. Physiother. Exerc..

[43] Paul L., Wood L., Behan W.M., Maclaren W.M. (1999). Demonstration of delayed recovery from fatiguing exercise in chronic fatigue syndrome. Eur. J. Neurol..

[44] Thomas M., Smith A. (2009). An investigation into the cognitive deficits associated with chronic fatigue syndrome. Open Neurol. J..

[45] Snell C.R., Stevens S.R., Davenport T.E., Van Ness J.M. (2013). Discriminative validity of metabolic and workload measurements to identify individuals with chronic fatigue syndrome. Phys. Ther..

[46] Cook D.B., Light A.R., Light K.C., Broderick G., Shields M.R., Dougherty R.J., Meyer J.D., Van Riper S., Stegner A.J., Ellingson L.D. (2017). Neural consequences of post-exertion malaise in Myalgic Encephalomyelitis/chronic fatigue syndrome. Brain Behav. Immun..

[47] Reynolds G.K., Lewis D.P., Richardson A.M., Lidbury B.A. (2014). Comorbidity of postural orthostatic tachycardia syndrome and chronic fatigue syndrome in an Australian cohort. J. Intern. Med..

[48] Lewis I., Pairman J., Spickett G., Newton J.L. (2013). Clinical characteristics of a novel subgroup of chronic fatigue syndrome patients with postural orthostatic tachycardia syndrome. J. Intern. Med..

[49] Miwa K., Inoue Y. (2018). The etiologic relation between disequilibrium and orthostatic intolerance in patients with Myalgic Encephalomyelitis (chronic fatigue syndrome). J. Cardiol..

[50] Kingdon C.C., Bowman E.W., Curran H., Nacul L., Lacerda E.M. (2018). Functional status and well-being in people with Myalgic Encephalomyelitis/chronic fatigue syndrome compared with people with Multiple Sclerosis and healthy controls. Pharmacoecon. Open.

[51] Komaroff A.L., Fagioli L.R., Doolittle T.H., Gandek B., Gleit M.A., Guerriero R.T., Kornish R.J., Ware N.C., Ware J.E., Bates D.W. (1996). Health status in patients with chronic fatigue syndrome and in general population and disease comparison groups. Am. J. Med..

[52] Lin J.S., Resch S.C., Brimmer D.J., Johnson A., Kennedy S., Burstein N., Simon C.J. (2011). The economic impact of chronic fatigue syndrome in Georgia: Direct and indirect costs. Cost Eff. Resour. Alloc..

[53] Nederlandse Vereniging voor Verzekeringsgeneeskunde (Dutch Association of Medical Insurance Physicians) (2018). Aanvulling op het Standpunt van de NVVG en GAV over het GR-advies ME/CVS: Multidisciplinaire Richtlijn CVS uit 2013 Blijft Onveranderd van Kracht en Prevaleert. https://www.nvvg.nl/nieuws/nieuws-nvvg/aanvulling-op-het-standpunt-van-de-nvvg-en-gav-over-het-gr-advies-mecvs/.

[54] Carruthers B.M., van de Sande M.I., de Meirleir K.L., Klimas N.G., Broderick G., Mitchell T. (2011). Myalgic encephalomyelitis: International consensus criteria. J. Intern. Med..

[55] Carruthers B.M., Jain A.K., de Meirleir K., Peterson D.L., Klimas N.G., Lerner A.M., Bested A.C., Flor-Henry P., Joshi P., Powles A.C.P. (2003). Myalgic encephalomyelitis/chronic fatigue syndrome: Clinical working case definition, diagnostic and treatment protocols. J. Chronic Fatigue Syndr..

